# Adenoviral hepatitis in two Nile crocodile (*Crocodylus niloticus*) hatchlings from South Africa

**DOI:** 10.4102/jsava.v90i0.1987

**Published:** 2019-11-26

**Authors:** Silke Pfitzer, Keagan J. Boustead, Jan H. Vorster, Lizette du Plessis, Louis J. la Grange

**Affiliations:** 1 Faculty of Agriculture and Natural Sciences, School of Biology and Environmental Sciences, University of Mpumalanga, Nelspruit, South Africa; 2 Valley Farm Animal Hospital, Pretoria, South Africa; 3 Vetdiagnostix Veterinary Pathology Services, Pietermaritzburg, South Africa; 4 Electron Microscope Unit, Department of Anatomy and Physiology, Faculty of Veterinary Science, University of Pretoria, Onderstepoort, South Africa; 5 Department of Agriculture, Rural Development, Land and Environmental Affairs, Veterinary Services, Nelspruit, South Africa

**Keywords:** adenovirus, hatchlings, Nile crocodile, *Crocodylus niloticus*, South Africa

## Abstract

Adenoviral infections may cause mild to severe morbidity or fatality in a large array of animal species. In crocodilians, hatchlings under 5 months of age are usually affected. However, there is a paucity of information on actual incidences in hatchlings originating from South Africa. Two cases of adenoviral hepatitis in crocodile hatchlings about 2 weeks old, bred on a commercial farm in South Africa, are described. Both hatchlings showed typical clinical signs of hepatitis. The identification of intranuclear inclusion bodies in the liver was used to differentiate between adenoviral hepatitis and chlamydial hepatitis. Although vertical transmission has never been proven in crocodiles, the young age of the affected hatchlings raises the possibility of vertical transmission. The lack of epidemiological information on adenoviral hepatitis in crocodiles highlights the need for further characterisation of the virus and targeted surveillance.

## Introduction

The family Adenoviridae consists of non-enveloped, icosahedral, double-stranded Deoxyribonucleic acid (DNA) viruses which replicate in the nuclei of cells (Davison, Benkȍ & Balázs 2003; Harrach et al. 2011; Marchang 2011). At least five genera are known to exist (Doszpoly et al. 2013; Harrach et al. 2011). Adenoviruses detected in squamate reptiles fall into the genus *Atadenovirus*, which has been detected in birds, ruminants and marsupials (Harrach et al. 2011; Wellehan et al. 2004). Genomic analysis and phylogenetic classification of adenoviruses from crocodiles are lacking.

In crocodiles, adenoviral infection usually affects hatchlings under 5 months of age (Huchzermeyer 2002, 2003). Clinical signs of disease include lethargy and anorexia and are sometimes associated with high mortality, especially during winter months when additional stress factors might also play a role in disease progression. Chronic adenoviral hepatitis can be a cause of stunted development (runting) of crocodiles (Huchzermeyer 2002). The liver is the most commonly affected organ; however, other organs may also be involved, including intestines, pancreas and lungs (Foggin 1987; Huchzermeyer 2003). The disease has frequently been detected in Zimbabwe (Foggin 1987), but the virus has only once been detected by electron microscopy in South Africa, from three crocodile hatchlings imported from Mozambique (Huchzermeyer 2003; Huchzermeyer, Gerdes & Putterill 1994b). The apparent low incidence in South Africa has been ascribed to a probable lack of contact with carriers of wild viruses (Huchzermeyer et al. 1994b). However, when raised in a stress-free environment with adequate biosecurity, the incidence and mortality rate associated with acute disease can be very low.

A differential diagnosis for acute hepatitis in Nile crocodile hatchlings is chlamydiosis, which has been previously observed in Nile crocodiles from South Africa. Macroscopically, chlamydiosis presents pathology similar to that observed with adenoviral infection (Huchzermeyer 2002; Huchzermeyer et al. 1994a; Huchzermeyer, Langelet & Putterill 2008). Adenoviral hepatitis can be differentiated from chlamydiosis on histological examination by the detection of intranuclear inclusion bodies (Huchzermeyer 2003).

## Case presentation

On 26 January 2016, a routine farm inspection was conducted on the crocodile farm by provincial veterinary authorities. The officers were informed of two hatchling mortalities that had occurred during the preceding evening. Further investigation revealed that another six hatchlings had died in the week prior to this event. No obvious gross pathology could be discerned, and the hatchling carcasses were transported to the premises of the responsible private veterinarian for post-mortem examination.

The commercial crocodile farm was situated in South Africa and has been in operation for more than 30 years. The farm housed approximately 25 000 crocodiles, including own breeding stock, hatchery and slaughter stock. A closed farming system was in place, and all hatchlings and slaughter stock were bred, hatched and raised on site. The annual mortality rate across all size and age groups was less than 2% of the total population.

Water was sourced from the local river that flows through the property. The river was inhabited by wild crocodiles and all water supplied to the hatchling houses was treated with ozone before entering the hatchling house. Approximately 5500 hatchlings were housed in the 480 m^2^ hothouse.

Both hatchlings were about 2 weeks of age. External examination showed the skin surfaces of both hatchlings to be intact and normal in colour. Eyes and nostrils were clear and no discharge was observed. Navel openings were fully closed and yolk sac resorption was partially complete without signs of inflammation. Similarly, cloacal openings appeared normal. Neck regions of both hatchlings appeared thin, suggestive of poor nutrition and/or starvation. Dentition and oral cavity appeared normal. The abdomen of one crocodile hatchling (B) was mildly distended.

The tonsillar tissue, gular valves, oesophagus, trachea and surrounding tissue showed no abnormalities in either of the hatchlings. Hatchling A had an accumulation of water in the throat and trachea consistent with drowning. All internal organs appeared normal in size, symmetry and colour. A partially absorbed yolk sac was observed in the abdomen and was within physiological parameters. Hatchling B displayed evidence of moderate hydrothorax with straw-coloured fluid surrounding the thoracic organs both inside and outside the confines of the post-pulmonary and post-hepatic transverse membranes. The lungs appeared normal. The liver was severely swollen. Disseminated petechial haemorrhages were seen in both hepatic lobes. All other organs appeared normal.

Fresh tissues collected were fixed in 10% phosphate-buffered formalin solution for at least 24 hours and routinely processed for histopathological examination according to standard techniques.

As a result of potential autolysis and increased rate of decomposition associated with the high humidity and temperature in the hothouse, no samples were collected for bacteriological culture.

**FIGURE 1:**
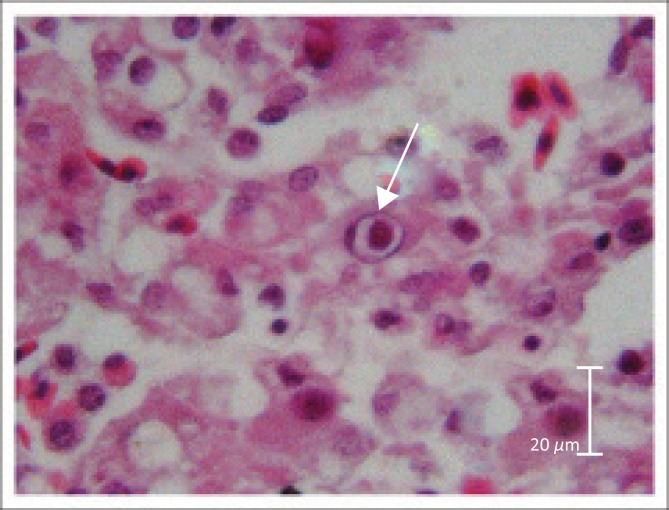
Basophilic viral inclusion body in the nucleus of a hepatocyte of one of the affected crocodile hatchlings (haematoxylin and eosin-stained section, magnification: 10×).

Histopathological examination in the case of crocodile A supported macroscopic findings and no significant histological lesions were identified in the lungs, fat body, heart or intestines. However, the liver tissue showed mild, diffuse congestion with mild to moderate diffuse degeneration and fatty changes of hepatocytes. Swelling of these hepatocytes resulted in mild sinusoidal occlusion. Mild, acute, multifocal hepatitis and single-cell necrosis was seen randomly scattered throughout the organ. A few larger foci of necrosis were also seen, which had small necrotic centres containing eosinophilic necrotic debris and necrotic cells. These necrotic foci were surrounded by rims of small numbers of macrophages. Many hepatocytes with numerous large intranuclear inclusions were observed. The nuclei were enlarged, with inclusions of varied sizes filling the entire nucleus. The inclusion bodies stained basophilic in haematoxylin eosin (HE)-stained sections (Figure 1).

Examination of the kidneys revealed mild diffuse congestion. The pancreas showed mild lymphoplasmacytic to granulomatous inflammation of the peri-pancreatic fat with similar intranuclear inclusions as observed in the liver.

In the case of crocodile B, the liver and fat displayed a pathology similar to that observed in crocodile A. Additionally, mild congestion of the spleen and mild, multifocal, chronic lymphocytic pericarditis and mild fibrosis of the pericardium could be identified.

These findings supported a diagnosis of necrotic viral hepatitis for both crocodiles and led to further investigation by electron microscopy.

**FIGURE 2:**
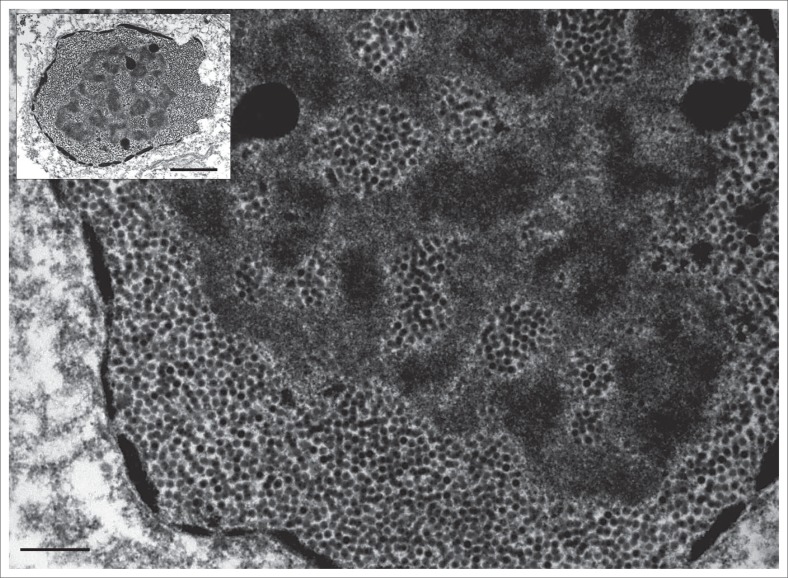
Electron micrograph displaying numerous adenovirus particles within the nucleus. Inset: Virus particles almost completely displacing nuclear material. Bar = 0.5*µ*m; inset bar = 2 *µ*m.

Using standard methods to prepare tissue samples for electron microscopy (Van den Bergh Weerman & Dingemans 1984), numerous virus particles in the size range of 70 nm – 90 nm were observed within the cell nuclei. In a number of nuclei, these particles displayed the crystalline appearance typically associated with adenovirus infection (Figure 2).

### Ethical considerations

This case describes post-mortem findings of crocodile hatchlings that died naturally on a crocodile farm. Post-mortem was carried out with the intention to investigate the cause of deaths and ameliorate further deaths if possible.

## Discussion

Based on macroscopic and histopathological findings, both mortalities could be ascribed to adenoviral hepatitis. The presence of numerous virus particles of crystalline appearance in the nuclei of cells as detected with electron microscopy supports this finding. Similar histopathological findings have been reported in two 8-month-old Nile crocodiles in which the infection was associated with enteritis (Jacobson, Gardiner & Foggin 1984). Although the hatchlings in our case were very young, both were emaciated. Anorexia can be a clinical sign of adenoviral hepatitis in crocodiles (Huchzermeyer 2002). In addition, the fact that one of the hatchlings showed signs of drowning may indicate that the disease progression rendered the animal too weak to escape from the water and it possibly drowned when other individuals were lying on top of it.

The small number of mortalities associated with this viral infection was also in agreement with previous literature which suggests that only some outbreaks led to mass mortalities, especially where stress is involved, and that subclinical infections are more prevalent (Foggin 1992; Huchzermeyer 2002). All of the hatchlings on this particular farm were kept indoors in conditions designed to minimise stress, such as artificially heated water to avoid thermal stress, and reduced noise and hide boards to reduce social stress. The stress-reduced environment might have contributed to the low mortality rate.

The young age of the hatchlings and the reported 2–18 weeks’ incubation period for adenoviral hepatitis (Huchzermeyer 2002) suggest that the infection might have originated within the egg. Vertical transmission of Adenoviridae has been reported in poultry (Grgić et al. 2006). Adenoviruses affecting reptiles may not be as host-specific as previously thought, and various other species could have played a role in the introduction of this virus into the hatchling house (Ascher et al. 2013; Ball et al. 2014). Ozone has been shown to be effective at inactivating adenovirus type 40 associated with human gastroenteritis at low concentrations (Thurston-Enriquez et al. 2005). The efficacy of the ozone treatment of the locally sourced river water in this case cannot be guaranteed and transmission via the river water cannot be completely ruled out.

## Conclusion

This, to the authors’ best knowledge, represents the first official report of adenoviral hepatitis from Nile crocodile hatchlings bred in South Africa. The only other reported case from South Africa involved three hatchlings imported from Mozambique (Huchzermeyer et al. 1994b). The hatchlings in this report may represent the youngest crocodiles reported to be infected with adenoviral hepatitis (C.M. Foggin, pers. comm., 01 March 2016).

Further virus characterisation and targeted surveillance of various age groups of Nile crocodiles both in captivity and in wild populations will provide a better understanding of actual virus prevalence, epidemiology and economic impact or potential conservation impact of this disease.
